# Virtual Patients on the Semantic Web: A Proof-of-Application Study

**DOI:** 10.2196/jmir.3933

**Published:** 2015-01-22

**Authors:** Eleni Dafli, Panagiotis Antoniou, Lazaros Ioannidis, Nicholas Dombros, David Topps, Panagiotis D Bamidis

**Affiliations:** ^1^Medical SchoolFaculty of Health SciencesAristotle University of ThessalonikiThessalonikiGreece; ^2^Open Knowledge Foundation (OKFN) GreeceThessalonikiGreece; ^3^Department of Family MedicineUniversity of CalgaryCalgary, ABCanada

**Keywords:** semantics, medical education, problem-based learning, data sharing, patient simulation, educational assessment

## Abstract

**Background:**

Virtual patients are interactive computer simulations that are increasingly used as learning activities in modern health care education, especially in teaching clinical decision making. A key challenge is how to retrieve and repurpose virtual patients as unique types of educational resources between different platforms because of the lack of standardized content-retrieving and repurposing mechanisms. Semantic Web technologies provide the capability, through structured information, for easy retrieval, reuse, repurposing, and exchange of virtual patients between different systems.

**Objective:**

An attempt to address this challenge has been made through the mEducator Best Practice Network, which provisioned frameworks for the discovery, retrieval, sharing, and reuse of medical educational resources. We have extended the OpenLabyrinth virtual patient authoring and deployment platform to facilitate the repurposing and retrieval of existing virtual patient material.

**Methods:**

A standalone Web distribution and Web interface, which contains an extension for the OpenLabyrinth virtual patient authoring system, was implemented. This extension was designed to semantically annotate virtual patients to facilitate intelligent searches, complex queries, and easy exchange between institutions. The OpenLabyrinth extension enables OpenLabyrinth authors to integrate and share virtual patient case metadata within the mEducator3.0 network. Evaluation included 3 successive steps: (1) expert reviews; (2) evaluation of the ability of health care professionals and medical students to create, share, and exchange virtual patients through specific scenarios in extended OpenLabyrinth (OLabX); and (3) evaluation of the repurposed learning objects that emerged from the procedure.

**Results:**

We evaluated 30 repurposed virtual patient cases. The evaluation, with a total of 98 participants, demonstrated the system’s main strength: the core repurposing capacity. The extensive metadata schema presentation facilitated user exploration and filtering of resources. Usability weaknesses were primarily related to standard computer applications’ ease of use provisions. Most evaluators provided positive feedback regarding educational experiences on both content and system usability. Evaluation results replicated across several independent evaluation events.

**Conclusions:**

The OpenLabyrinth extension, as part of the semantic mEducator3.0 approach, is a virtual patient sharing approach that builds on a collection of Semantic Web services and federates existing sources of clinical and educational data. It is an effective sharing tool for virtual patients and has been merged into the next version of the app (OpenLabyrinth 3.3). Such tool extensions may enhance the medical education arsenal with capacities of creating simulation/game-based learning episodes, massive open online courses, curricular transformations, and a future robust infrastructure for enabling mobile learning.

## Introduction

Contemporary medical education has progressively extended into a wide variety of learning resources and domain-specific educational activities that have become more and more digitized [1]. The inherent driving force behind this is the need for worldwide access to clinical skills development, independent of time and place [2]. Much of this potential of information and communication technology (ICT) in medical education is due to the advancement of Web technology and the development of interactive learning environments with immediate, content-related feedback [3].

Modern medical education is largely based on case-based or problem-based learning (CBL/PBL) and other small-group instructional models [4,5]. Virtual patients, defined as “interactive computer simulations of real-life clinical scenarios for the purpose of health care and medical training, education or assessment” [6], have become one of the most commonly used CBL/PBL types in modern medical education [7] and have proved to be especially useful in teaching clinical decision making [8].

Moreover, Web-based virtual patients, unlike real patient practice, are inherently repeatable [9] and offer few limitations with respect to time, place, and failure-safe practice of clinical skills. Medical students have the opportunity to practice on any disease that may be encountered later in clinical practice, even rare or highly risky cases [10]. The reproducibility and capacity for standardized, validated assessments have made virtual patients an important and effective tool in modern medical education [11-13].

There is a worldwide trend to develop virtual patients and many academic institutions are working toward this goal [14]. However, some of their main disadvantages are that they are expensive and resource-intensive to develop [10]. Currently, few academic institutions can afford to dedicate resources for full-scale virtual patient development, thus facilitating the creation of online virtual communities where virtual patients can be shared as educational resources [6]. Open educational resource (OER) advances and innovative Web technologies have boosted content sharing and retrieval over the past few years. Web 2.0 encouraged a more human-centered approach to interactivity, with much support for group interaction, and fostered a greater sense of community in a potentially “cold” social (learning) environment [15].

To integrate the aforementioned evolutions toward a more sharable, searchable, and repurposable virtual patient paradigm, 3 aspects are being addressed: (1) the Semantic Web approach for annotating and consuming content, (2) the formulation of stable and standardized platforms for developing and deploying virtual patients, and (3) a cohort of proof-of-application studies in the form of formative and summative evaluation studies.

In the first aspect, the potential of Web 3.0, the Semantic Web, has added more dimensions beyond traditional Web services concerning education and research with a greater capacity for cognitive processing of information [16]. A primary feature of Web 3.0 is the use of metadata: “structured information that describes, explains, locates, or otherwise makes it easier to retrieve, use, or manage an information resource” [16].

Exploring this further with virtual patients, Semantic Web technology provides an opportunity to structure information within the virtual patients, so as to enable easy retrieval, reuse, and exchange of cases between different systems. Many European academic institutions use their own virtual patient authoring systems to deliver virtual patient cases for their own curricula; therefore, educational silos are formed because of this difficulty of sharing virtual patients across different platforms. The MedBiquitous Virtual Patient ANSI/MEDBIQ VP.10.1-2010 is a technical standard that enabled the development of global repositories of virtual patients [17]. Virtual patient cases in MedBiquitous standard format can be exchanged across systems or be exported in a MedBiquitous Virtual Patient package [17]. However, search and retrieval of specific content can be problematic when utilizing existing keyword-based searches. These often miss relevant cases or retrieve irrelevant information; for example, by contextual differences in the use of a keyword or where synonyms of the search term are employed. Moreover, with regard to the meaningful extraction of information, human browsing and reading is required to manually extract relevant information about medical resources. Without semantic metadata, virtual patients lack capacity to expose or consume meaningful information with other data consumers or providers. Semantic annotations allow context, structure, and content descriptions of virtual patients’ data, providing completely new possibilities: robust and reliable searches, complex queries, and improved virtual patient exchange [18]. The mEducator Best Practice Network (BPN), funded by the European Commission within the eContentplus programme, is the first project globally to use Semantic Web services and linked open data [19] to not only federate different educational platforms, but also to publish educational resource descriptions on the Web of data. This project, after analyzing the use of existing standards and reference models in the e-learning field, aimed to develop mechanisms and best practices for discovering, retrieving, sharing, and reusing medical educational resources [20,21]. That overall goal incorporates the challenge of linking virtual patients with other resources available on the linked open data cloud and the Semantic Web, thereby paving new ways of using and exploiting virtual patients [22].

In the second aspect, that of standardized platforms for virtual patient creation and deployment, 1 of the most popular virtual patient authoring and delivering systems, OpenLabyrinth, is widely used across various academic institutions because it consists of an open source toolset that allows the creation and delivery of a wide range of pathway-based educational activities [23] with an easy-to-use, code-free interface [24]. The user-friendly interface, the level of Information Technology knowledge required for the case developers, and its compliance to the MedBiquitous standard have made OpenLabyrinth a popular virtual patient authoring tool for medical education [25]. However, despite its widespread use for virtual patient deployment, OpenLabyrinth offers a limited description of virtual patient resources and no resource search mechanisms. The core version of OpenLabyrinth does not offer a prominent, clear, and standards-based solution for the seamless sharing of virtual patients. The mEducator extended OpenLabyrinth module, OLabX, aimed to address these needs by applying the mEducator schema. OLabX has been developed to allow virtual patients to be described, shared, and semantically searched. OLabX offers easy virtual patient retrieval, sharing, and repurposing through a standards-based infrastructure that enables the sharing of this state-of-the-art digital medical educational content among medical educators and students of higher academic institutions.

In the final aspect, that of the evaluation and validation of virtual patients as efficient standalone learning tools and teaching aids in different modalities, things are recently picking up speed. Even though a lot of effort has been put in the area of virtual patients’ development, description, and sharing, few studies are referred to a comprehensive evaluation of virtual patient authoring systems and efficacy of integration of electronic virtual patients in the medical curriculum. Medical students seem to embrace teaching and assessment through virtual patients and that is a prerequisite for virtual patients to be adopted widely [26]. The pilot evaluation of Web-SP, another Web-based virtual patient authoring tool, resulted in positive conclusions regarding the creation, management, and evaluation of Web-based virtual patient cases, but further studies looking at the learning outcomes, critical thinking, and patient management are required [27]. The trend of pilot evaluations of virtual patient-based learning episodes has been followed by a recent formal randomized controlled trial study [28] that aimed to investigate the efficacy of dynamic Web-based virtual patients in PBL sessions (dynamic PBL), the results were highly encouraging. Compared to a linear PBL group, the dynamic PBL groups’ performance was better and this difference was statistically significant for all questions related to dynamic PBL.

This work is presented in 3 parts: (1) the rationale and basic principles that govern the transition to the Semantic Web, (2) a presentation of the architecture for extending OpenLabyrinth, a commonly used tool for virtual patients, introducing another free and open source distribution mechanism, and (3) an initial assessment of the feedback received from pilot evaluations of the tool.

## Methods

### Overview

The scope of this methodology section is twofold. On the technology front, we present a brief overview of how we extended the OpenLabyrinth platform using the mEducator metadata schema thereby creating OLabX. The mEducator OpenLabyrinth extension, OLabX, has been developed to allow virtual patients to be created, described, shared, searched, and easily repurposed. OLabX has enriched the global metadata describing a Labyrinth by applying the mEducator schema through the existing OpenLabyrinth global metadata editor. Therefore, a new database schema and interface has been proposed to implement the classes and properties defined by the mEducator schema. On the educational front, we describe the evaluation of the efficacy of the OLabX platform as a repurposing and authoring platform. This was done through a number of user scenarios and clinical tasks, as well as with formal system usability testing. The second part of the evaluation consists of assessing the repurposed content itself through previously verified and tested evaluation instruments [29].

### The mEducator Metadata Schema

The mEducator schema defines the metadata accompanying an educational resource (eg, a virtual patient case) [20]. This metadata, often highly structured, is designed to support specific functions [30]. It also affords repurposing of educational resources [31]. The metadata schema is based on the established standard Health Care Learning Object Metadata [32], which is an extension of the Institute of Electrical and Electronics Engineers Learning Object Metadata, a more general standard [33]. The mEducator schema consists of 10 mandatory fields and a number of optional fields as demonstrated in Table 1.

A widget for searching SNOMED and Medical Subject Headings (MeSH) clinical terms via the National Center for Biomedical Ontology (NCBO) BioPortal platform [34] has also been implemented for coding consistency with the mEducator schema [35]. mEducator 3.0 implements open linked education functionality using Semantic Web services. As mentioned previously, an extension (OLabX) that builds on the mEducator schema has been developed for OpenLabyrinth. The extension is intended to make mEducator 3.0 more accessible to new and current users of this system and overcome the lack of standardized virtual patient sharing mechanisms [36].

**Table 1:** Overview of the mEducator metadata schema.

Mandatory fields	Data type	Optional fields (nonexhaustive list)	Data type
Identifier	Number	Educational objectives	Free text
Title	Free text	Educational outcomes	Option from code list
Creator	Free text	Assessment methods	Free text
Intellectual property rights license	Option from code list	Educational context	Free text
Language of the resource	Option from code list	Technical description	Free text
Language of the metadata	Option from code list	Discipline	Free text
Creation date	Option from date picker	Discipline speciality	Free text
Metadata creation date	Option from date picker		
Keywords	Free text		
Description	Free text		

### Architecture of the OLabX Application

OpenLabyrinth is an open source, Web-based, activity modeling system that allows users to build interactive “game-informed” educational activities, such as virtual patients, simulations, games, mazes, and algorithms, and is used by various medical academic institutions [37]. OpenLabyrinth is a powerful platform for delivering game-based learning exercises, which have been shown to be effective for learners [38]. The Web-based nature of the tool means that it is platform-independent from the user’s perspective, and easily accessible from any computer hardware through a browser, making it ideal for use in an educational environment [38]. Thus, it offers a convenient way for trialing new learner-centric pedagogic approaches such as those involving PBL paradigms.

Although the core version of OpenLabyrinth does not support advanced repository functionalities, OLabX, the mEducator OpenLabyrinth extension, enriches the metadata, describing a virtual patient by applying the mEducator schema on the existing metadata editor. This allows for enhanced learning scenarios to be facilitated, such as the following scenario: A medical student interested in acute myocardial infarction would like to access a relevant virtual patient case. Using her device, she would access the OLabX app through a Web browser. She would then use the app’s search facility to submit a query for acute myocardial infarction. In the previous version of the app, only virtual patient cases with “acute,” “myocardial,” and/or “infarction” keywords would appear, whereas the app is now able to search through synonymous keywords and related medical areas. “Heart attack” and “cardiology” would also yield results when such a search was performed. Given a relatively large amount of available virtual patient cases, this saves the student from browsing the collection manually or entering the alternative keywords herself. Going a step further, OLabX is now able to store her preferences in content and intelligently suggest similar content coming in other formats within the mEducator-enabled network of resources. This capability stems from the network nature of the Resource Description Framework (RDF) metadata, which can be connected with existing medical vocabularies and other resources described as linked data. This architecture also considers future enabled scenarios in which search engines not only read the page-embedded RDF content, but also safely infer and index relationships between resources that are not otherwise explicitly linked.

**Figure 1:**
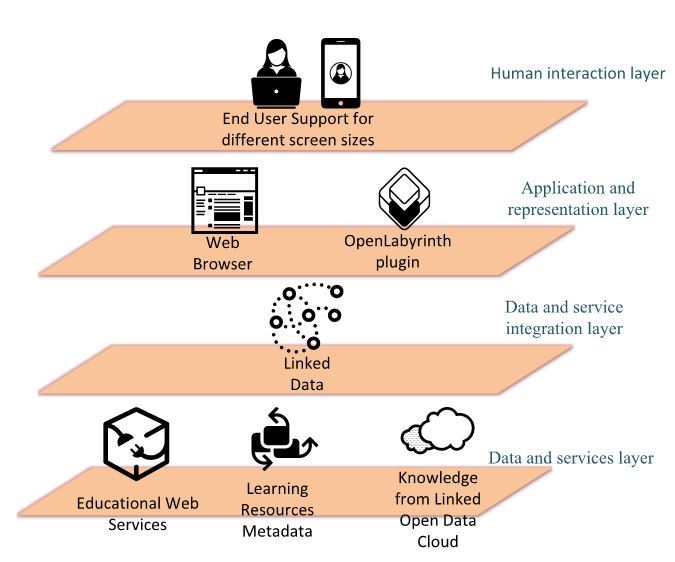
Overview of the mEducator interaction architecture.

That kind of scenario is supported by the overall interaction architecture that is demonstrated in Figure 1. This includes 3 layers [39]. The first (lowest), the (Web) data and service layer, consists of the available Learning Resource Metadata (LRM), Web services, and data sources (such as the linked data cloud). In the aforementioned example, at this level disambiguated, contextualized “bare bones” search terms from the previous layer would be consumed by the Web services to be compared against the linked open data cloud. Singular or multiple search terms, contextual synonyms, or contextual excluded terms formulate a complex search in the open data cloud. Then the relevant virtual patient cases would be discovered through their published resource metadata that the system is able to discover in this layer. The second (moving higher) is the data and service integration layer where the whole linked data infrastructure of OLabX is realized. The data and service integration layer is based on the linked services approach [40] using 2 repositories, iServe and SmartLink, and by exposing application program interfaces, which can be used to access the services programmatically. At this level, in the previous example, the “parsing,” disambiguation, and contextualization of the search terms for acute myocardial infarction would occur to provide relevant data to the data and service layer for its search and retrieval facilities. The third layer, the app and representation layer, is a straightforward user interface front end and OpenLabyrinth plug-in that maintains the necessary provisions for seamless integration with the rest of the OpenLabyrinth platform. The search user interface and the process for invoking the OpenLabyrinth virtual patient Web player are all included in this layer, which is also what is visible to the end user through the device on which the virtual patient episode is run. The app can be accessed and used conveniently by any standards-compliant Web browser. This facet of the OpenLabyrinth extension, along with a template to allow other systems to embrace the mEducator schema, affords semantic description of medical educational resources.

### OLabX Functionalities

**Figure 2:**
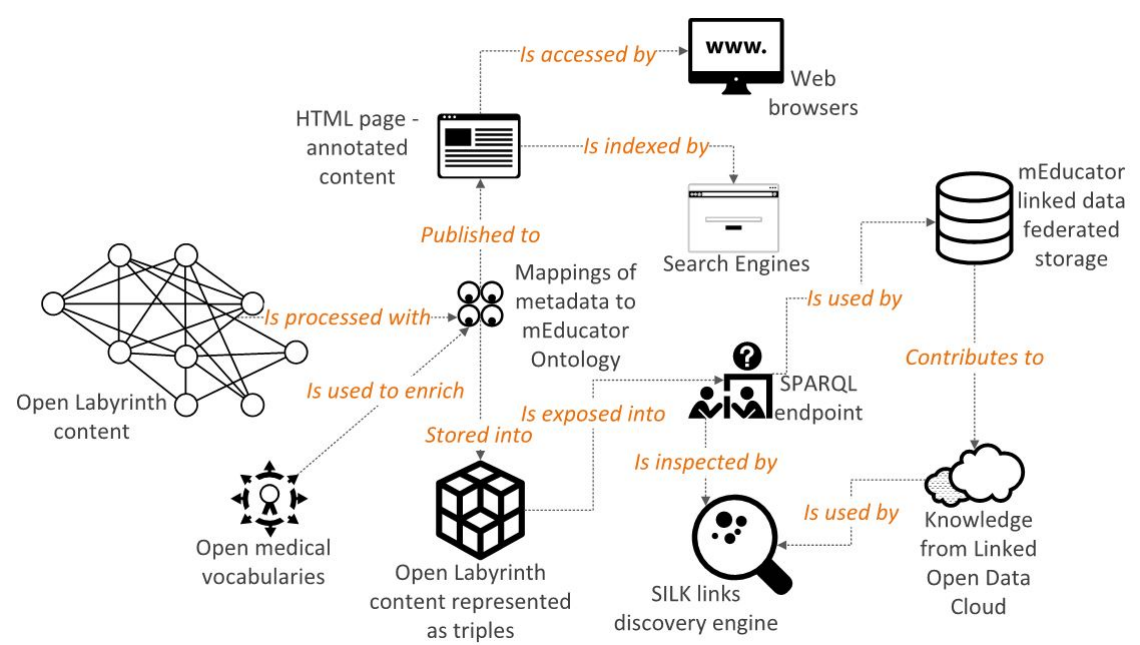
Overview of the mEducator3.0 OpenLabyrinth module.

The previously described architecture enables (1) resource queries across distributed and heterogeneous learning content management systems, whereas query results are automatically converted into the RDF, (2) storage of the metadata in an RDF store, and (3) metadata enrichment (eg, from SNOMED and PubMed) (Figure 2). The resulting mEducator metadata accompanying a virtual patient case are editable and searchable by all authorized users.

In the OpenLabyrinth case editor, a new field set was created including the mEducator metadata, namely “mEducator Metadata Entry.” The original OpenLabyrinth metadata description schema was extended to be consistent with the mEducator metadata schema. This schema extension allowed the labyrinth authors to use existing, standardized medical taxonomies in a structured way. For instance, the mEducator ontology property named “subject” allowed the editor to select values from an autocomplete list that corresponds to terms from ontologies registered in the NCBO BioPortal platform [34]. This metadata annotation is important for searching among a large set of learning resources. The OpenLabyrinth database was extended, creating 2 kinds of tables: the first describing the mEducator classes and properties and the second linking the mEducator properties values for each virtual patient case. This change is nonintrusive, meaning it does not cause any administrative hassles and is reversible.

The mEducator schema also allows the repurposing of a case in a different educational context. A virtual patient can be repurposed to a different language, to a different culture, for a different pedagogical approach, for a different educational level, for a different discipline, to a different content type, and to a different educational technology [31,41]. Every medical educator is engaged with educational content transformation to some extent and repurposing becomes a necessary and common procedure in medical education. The semantic extension of OpenLabyrinth offers the opportunity of effective repurposing and reuse of a virtual patient case by medical educators and students as well. Furthermore, export-import and duplication functionalities have been extended according to the mEducator schema requirements. The original OpenLabyrinth exports the whole labyrinth in the MedBiquitous Virtual Patient data format [17]. In addition, a new feature has been implemented that allows exporting or importing the labyrinth’s metadata in the mEducator metadata exchange format. It also adds support for duplicating a labyrinth together with its accompanying mEducator metadata, extending the original duplication functionality. Additionally, it facilitates the creation of a social hierarchy of linked repurposed resources in a parent-child approach, thereby allowing for a whole network of linked resources for which the resource creators are linked with repurposing actions (who is repurposed from whom).

### Evaluation Methodology

For evaluation of the OpenLabyrinth extension, a detailed test plan was designed. This included the identification of the overall objectives of the evaluation. The main point of the semantic extension, apart from virtual patient creation, was the sharing and repurposing of cases. Thus, it was important to evaluate system usability and efficacy as a repurposing tool. Moreover, an additional goal of this evaluation would be to provide an assessment of the output quality of the repurposed virtual patients created. Additionally, the overall evaluation was assessed by 2 expert reviewers whose sole aim was to provide meta-feedback for the whole process.

**Figure 3:**
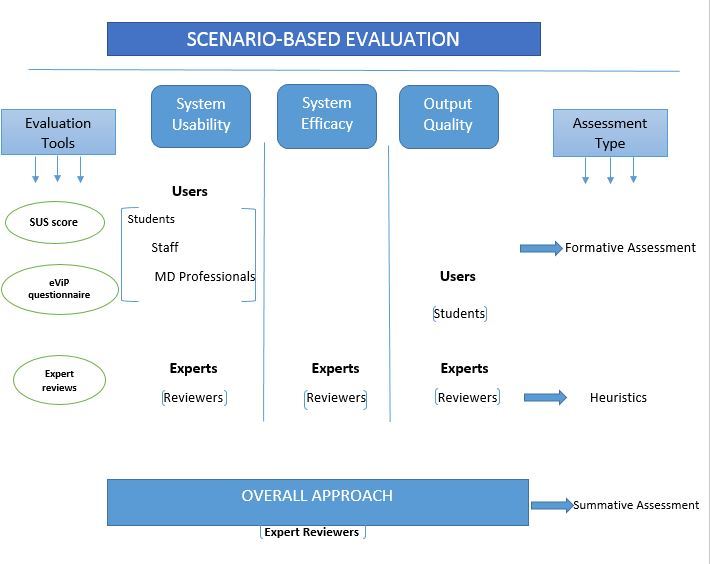
Methodology of the evaluation process.

The evaluation methodology included formative assessment through user studies with testing scenarios and questionnaires, heuristic assessment through expert reviews, and summative assessment through the overall evaluation of the process. Formative assessment was used in the evaluation of the system usability and the quality of the output using formal instruments such as the System Usability Scale (SUS) questionnaire [42] and the student questionnaire from the previously validated electronic virtual patients (eViP) evaluation tool kit [43]. Expert reviews were used in all aspects of the evaluation and in the overall assessment of the process. The overall evaluation strategy is described in Figure 3.

### Scenario-Based Evaluation Approach

The overall evaluation strategy was based on use case scenarios. Expert reviewers or formative assessment user groups acted as evaluators. User tests were performed according to the scenarios for sharing, searching, retrieving, and repurposing content. Testing groups included educators, health care professionals, and students.

The scenarios covered specific tasks: (1) virtual patient sharing, (2) virtual patient search and retrieval, and (3) virtual patient repurposing. The scenarios were initially presented to participants in a scheduled hands-on workshop organized in Vilnius, Lithuania, during the evaluation phase of the mEducator project. The purpose of that workshop was to familiarize participants with the creation, searching, and repurposing of resources within the environment of OLabX. The user sample consisted of approximately 12 health professionals from different specialties, including educators, students, and health care policy makers under the guidance of trained workshop facilitators.

The second part of the evaluation took place at the Medical School of the Aristotle University of Thessaloniki (AUTH), Thessaloniki, Greece, and was divided into 2 phases. The first phase included 2 categories of evaluators at the same time: (1) the team of medical educators that participated in the design and implementation process of virtual patient cases by OLabX after training workshops on the use of the platform and the rationale of Semantic Web annotations and (2) undergraduate medical students, randomly selected by a random student identification number generator from the final year cohort of the undergraduate curriculum, after suitable training in OpenLabX use. In total, 33 evaluators, 20 medical teachers, and 13 medical students of AUTH participated in this phase. The second phase included 48 undergraduate medical students randomly selected from a cohort of 120 students attending the undergraduate elective course on medical education. These students used the eViP questionnaire to assess the quality of repurposed virtual patients.

In both phases, the evaluation scenarios had a similar structure as the scenario used in the Užkrečiamųjų ligų ir AIDS centras (ULAC; Centre for Communicable Diseases and AIDS) workshop mentioned previously. Details of these scenarios are summarized in Table 2.

**Table 2:** Testing scenarios for the assessment of the OLabX virtual patient creation, search, retrieval, and repurposing platform.

User tests	Scenario 1	Scenario 2	Scenario 3
Aim	Familiarize users with the virtual patient creation process	Familiarize users with searching of resources	Familiarize users with virtual patient repurposing
Objectives	Show users how to log in; explain the basic functions involved in virtual patient creation; explain the notion of virtual patient metadata	Show users how to search for virtual patients, in comparison to simple search engines; explain the role of basic search attributes; explain how metadata enhances search functions	Show users the role of repurposing; explain intellectual property rights (IPR) metadata; demonstrate completion of assessment metadata
Expected learning outcomes	(1) Login to OLabX successfully, (2) understand the notion of metadata to trace it at a later stage, (3) enter the appropriate metadata using the system forms, (4) save the metadata in OLabX, and (5) visualize the entered metadata	(1) Specify the search attributes, (2) perform the search, and (3) appreciate and analyze the obtained results	(1) Perform case repurposing, (2) specify the IPR metadata for their own resources, (3) correctly enter repurposing metadata, (4) search for repurposed resources, and (5) appreciate and analyze the obtained results

For usability and efficacy testing by expert users, the same scenario-based approach was used. Experts were provided with a more open list of tasks:

1. Perform search(es)
2. Study and evaluate the results comparing to the search goals
3. Refine the search terms to perform new searches
4. Decide on which resources to inspect/download
5. Decide on the necessity of further searches
6. Decide on which resources to use later

The only deviation from this scenario-based approach was in the heuristic assessment that was performed by the overall evaluation expert reviewers. In their case, an observer’s role was employed and they were allowed to express their feedback, in an open-ended qualitative manner, regarding the overall evaluation process.

### System Usability Assessment

The first axis of system usability assessment consisted of a formative user-group usability survey and the standard SUS questionnaire.

The SUS questionnaire assesses the overall usability of the system. It is a 10-item questionnaire with 5 Likert-type response options ranging from the extreme positive (strongly agree) to the extreme negative (strongly disagree) and with half of the questions phrased in negative assertions to avoid bias [42]. It is a “quick and dirty” usability measurement instrument that produces a standardized score ranging from 0 to 100 that can be used to directly compare the usability between systems [44]. The results from both groups were collected and normalized into the standard percentile rank that facilitates comparisons between systems.

The second axis of system usability testing was conducted through an expert review of the system. The main directive that was provided to the reviewer was to assess the usability of the user interface and the overall look and feel of the platform within the scope of creating content in the OLabX platform. Specifically, the heuristic qualifiers that the reviewer was asked to check the system against, were the following:

1. The user interface is intuitive and facilitates content creation.
2. The system provides clear feedback to the user about her/his actions.
3. The system provides a level of accessibility and automation options that are expected of contemporary Web-based platforms.

### Efficacy Assessment

The efficacy evaluation task was performed by 1 medical pedagogy specialist online and remotely. The reviewer explored the OLabX mEducator 3.0 instantiation freely, but trialed a selection of search terms from the following list: dermatology, cardiology, tumor, cancer, neoplasm, simulation, image, and clinical case. For search and retrieval, the expert was asked to perform the operations on the system previously mentioned. The expert’s task was to comment on the use of the system using several heuristic qualifiers and an overall assessment of the strengths and weakness of the platform’s efficacy along with open-ended recommendations and comments. The specific qualifiers used by the expert reviews for assessing the efficacy of the OLabX platform are listed in Textbox 1.

**Specific qualifiers for assessing the OLabX platform.**

Formulation (expressing the search):

1. User is provided access to appropriate resources in library and collections.
2. Structured fields for limiting the source are used (year, media, language, etc).
3. Entered phrases are recognized.
4. Variants such as differences in casing, partial matches, etc, are allowed.
5. The size of the result set is controlled.

Initiation of action (launching the search):

1. Explicit actions are included (consistently labeled, located, sized, and colored buttons).
2. Implicit actions are included (changing a parameter immediately produces new sets of results).

Review of results (reading messages and outcomes):

1. Explanatory messages are presented.
2. Overview of results and preview of items are viewed.
3. Visualizations can be manipulated.
4. The size of the result set and shown metadata fields can be adjusted.
5. Sequencing can be changed.
6. Clustering can be explored.
7. Selected items can be examined.

Refinement (formulating the next step):

1. Meaningful messages guiding the user in progressive refinement are provided.
2. Parameters are easy to change.
3. Feedback relevance is explored.

Use (compiling or disseminating insight):

1. Queries, settings, and results are allowed to be saved and annotated, emailed, or used as input for other tools.

### Output Quality Assessment

Evaluating how medical students use repurposed virtual patients is crucial for the effective development and exploitation of repurposed educational resources [45]. For the output quality evaluation, an evaluation instrument developed and validated during the eViP European project for the creation and sharing of virtual patients was used [43].

This tool is for the evaluation of students’ experience with virtual patients, focusing on the development of clinical reasoning skills. This questionnaire contains 7 subsets totaling 14 items. Likert scale questions were used in this questionnaire with their statements based on attitudes and cognitive activities. These questions play an important role in clinical reasoning skills. Additionally, a few open-ended questions were included. This instrument was administered to 48 undergraduate students after they encountered a group of repurposed virtual patients.

### Overall Evaluation

Two observer expert review reports of the general performance were included in this evaluation process. The experts were familiar with the project, but they were not involved in the design or implementation tasks of OLabX. Their role was to act as observers in mEducator 3.0 OpenLabyrinth workshops and submit to the evaluation organizers an overall review for the workshops and the OLabX performance. Their feedback would provide a summative meta-evaluation of this diverse process of assessment to identify weaknesses and blind spots in the process itself. Given the scope and purpose of the required feedback, the reviewers were allowed to express this feedback in an open-ended fashion according to their overall experience and project-specific impressions.

## Results

### Usability Evaluation

**Figure 4:**
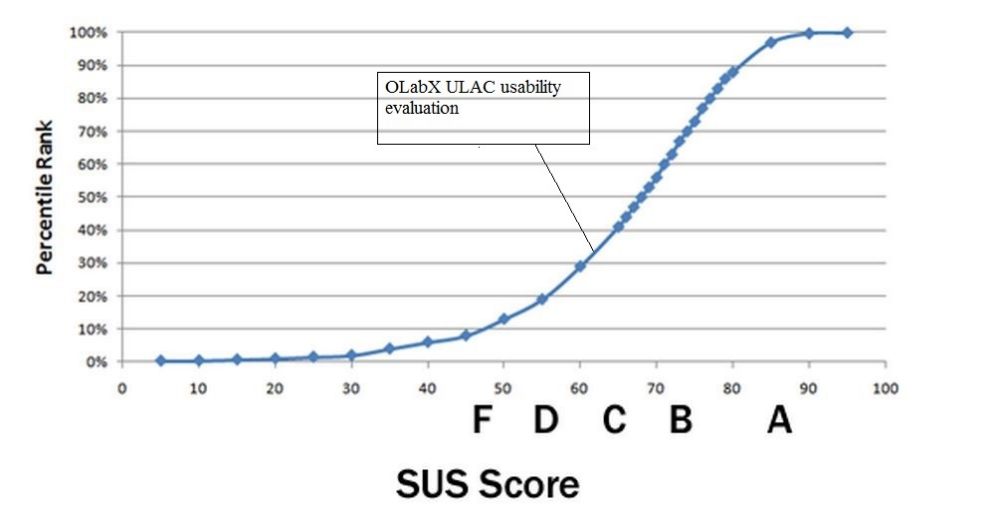
Formative assessment results from the system usability evaluation from users (MD professionals), placing the OLabX higher than 30% of all products tested.

The following are the OLabX testing results from the ULAC workshop. The raw SUS score from all 12 questionnaires was a 60.8 placing the OLabX application higher than approximately 30% of all products tested. The graph presented in Figure 4 shows the percentile rank associated with the SUS score and letter grades.

**Figure 5:**
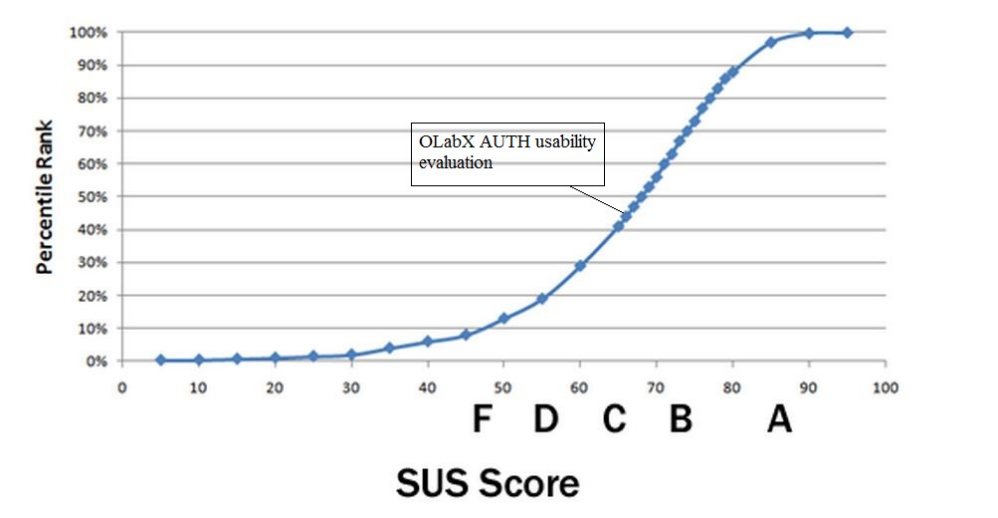
Formative assessment results from the system usability evaluation from users (students and staff), placing the OLabX higher than 40% of all products tested.

Next presented are the OLabX testing results from the AUTH workshop. The raw SUS score from all 33 questionnaires was a 65.83, placing the OLabX application higher than 40% of all products tested. Figure 5 shows the percentile rank associated with the SUS score and letter grades.

These scores were the trigger for the exploratory expert reviews to both verify the usability results from the SUS questionnaires, but primarily to identify areas of improvement required for placing the OLabX application in a competitive place in the overall SUS classification of products. Presented in Table 3 are the results of the heuristic evaluations of the 2 expert reviewers regarding the usability of the system.

From the feedback received from the expert reviewers, the main usability weaknesses were not related to the core OLabX functionality, but they were primarily attributed to standard computer applications’ ease of use provisions. These provisions were indeed absent because the focus was on implementing core functionality extensions at this point in the development of the platform. This could be considered the main reason that the OLabX platform did not score competitively in the SUS hierarchy of products.

**Table 3:** Heuristic usability feedback from expert reviewers.

Feedback		Recommendations/Solutions
**The user interface is intuitive and facilitates content creation.**		
	Links should perhaps be “link colored”	Use common interface conventions of Web services
	All tabs should be visible by default	Provide an overview of the creation process steps at start
**The system provides clear feedback to the user about her/his actions**		
	During the test the error messages after submitting a resource were not useful to common user	Ensure the readability and relevance of the error messages to the user
**The system provides a level of accessibility and automation options that are expected of contemporary Web-based platforms**		
	Date should be automatically inserted	Use automatic calendar functionalities when possible
	No provisions for multilingual keywords	Multilingual support should be implemented

### Efficacy Evaluation

The results of the efficacy evaluation of the OLabX platform from the expert reviewers are summarized in Table 4.

Although most responses touched on usability, these concern core functionality issues that directly impact efficacy. Because no formative assessment has been conducted, these platform-specific notes, although touching on usability issues, are the best indicators for the efficacy of the platform for creating repurposed virtual patient content.

**Table 4:** Heuristic efficacy evaluation feedback from expert reviewers.

Phase of search process	Strengths	Weaknesses
Expressing the search	Letter casing and partial matches allowed; IPR and/or language can be used	No content overview; no real-time phrase recognition suggestions; no control over the size of results
Launching the search	Some implicit actions are included	Some explicit actions are missing (back to results, new search); a complete schema is problematic for locating key functions on the interface; sorting options should be visible by default
Reading messages and outcomes	Textual preview of results	No search progress info; no summary; no control over size of result set; no control over sequencing/order
Formulating the next step		System provides no automated feedback/support for refining the search
Compiling or disseminating insight		System provides no corresponding functionalities
Comments and recommendations	The extensive metadata schema presentation in the interface invites user to exploring the filtering of resources	The lack of support in terms of interface usability for user guidance needs to be corrected to fully exploit the richness of the schema

### Output Quality Evaluation

#### Overview

**Figure 6:**
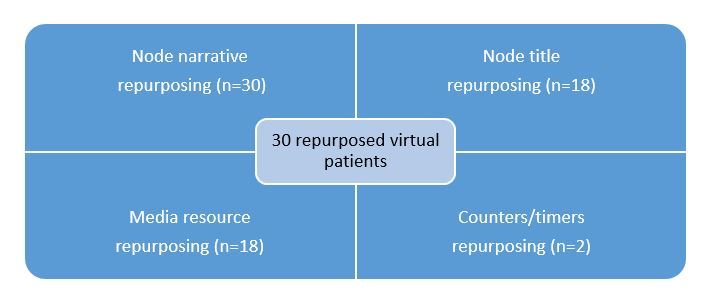
Repurposing types of virtual patients produced during the evaluation scenarios of OLabX.

From the preceding descriptions of the scenarios, quite a few repurposing attempts were conducted during the evaluation of OLabX. Overall, approximately 30 repurposed virtual patients were produced, with different aspects of repurposing applied during the process. More specifically, repurposing could refer to modifying at least the virtual patient node narrative and the media content of the virtual patient or the counter/timer measures. Figure 6 illustrates the overall output production of repurposed virtual patients during the evaluation scenarios.

A total of 48 undergraduate students were invited to choose approximately 5 from this pool of 30 repurposed virtual patient cases and then use the eViP questionnaire to provide feedback on the quality of the content. The following are the results from the repurposed virtual patient evaluation (Table 5). Most evaluators provided positive statements regarding the virtual patient content when the educational experiences with both the content and the system were considered. The eViP questionnaire also contained several open-ended questions, which are summarized subsequently.

**Table 5:** Results from the evaluation of the output quality (repurposed virtual patients) from users as part of the formative assessment.

Questionnaire subset theme and questions		N	N/A	Rating,^a^ n				
				1	2	3	4	5
**1. Authenticity of patient encounter and the consultation**								
	Q1. While working on this case, I felt I had to make the same decisions a doctor would make in real life.	48	2	0	2	6	36	2
	Q2. While working on this case, I felt I was the doctor caring for this patient.	48	2	0	3	12	25	6
**2. Professional approach in the consultation**								
	Q3. While working through this case, I was actively engaged in gathering the information I needed (eg, history questions, physical exams, laboratory tests) to characterize the patient’s problem.	48	2	2	5	11	16	12
	Q4. While working through this case, I was actively engaged in revising my initial image of the patient’s problem as new information became available.	48	3	0	3	10	27	5
	Q5. While working through this case, I was actively engaged in creating a short summary of the patient’s problem using medical terms.	48	2	0	4	11	25	6
	Q6. While working through this case, I was actively engaged in thinking about which findings supported or refuted each diagnosis in my differential diagnosis.	48	3	0	2	9	24	10
**3. Coaching during consultation**								
	Q7. I felt that the case was at the appropriate level of difficulty for my level of training.	48	5	5	15	10	10	3
	Q8. The questions I was asked while working through this case were helpful in enhancing my diagnostic reasoning in this case.	48	2	0	1	11	23	11
	Q9. The feedback I received was helpful in enhancing my diagnostic reasoning in this case.	48	3	1	6	16	15	7
**4. Learning effect of consultation**								
	Q10. After completing this case, I feel better prepared to confirm a diagnosis and exclude differential diagnoses in a real-life patient with this complaint.	48	2	7	2	14	6	7
	Q11. After completing this case, I feel better prepared to care for a real-life patient with this complaint.	48	3	1	4	9	21	10
**5. Overall judgment of case workup**								
	Q12. Overall, working through this case was a worthwhile learning experience.	48	3	0	0	7	24	14

^a^ 1=strongly disagree; 2=disagree; 3=neutral; 4=agree; 5=strongly agree.

#### What Are the Special Strengths of the Case?

Most participants pointed out that repurposed virtual patients were well constructed and delivered in ways that will elicit clinical reasoning skills in trainees. They allow for cognitive errors, through which the trainees learn without impact on a real patient. Training, development of critical thinking, and “virtual practice” before seeing real patients were the next popular features of repurposed virtual patients mentioned by the students. Better anxiety management in a safe environment was a popular answer, whereas a decision-making exercise and self-assessment were reported as advantages of great importance. They mentioned that these virtual patients contribute to the improvement of medical education and increase motivation for learning.

#### What Are the Special Weaknesses of the Case?

The most popular answer to this question was the statement that it is all about virtual patients and not real ones and virtual patients cannot replace the contact with real patients. Furthermore, stressful conditions that exist in real life are not fully reproduced with these virtual patients. Other comments sporadically mentioned were that questions had too few and too specific response answers.

#### Do You Have Additional Comments?

The additional comments conclusively included a summary of the students’ evaluations. A common comment referred to the fact that OLabX offers, through repurposing, the opportunity of virtual patient adjustment to the student’s level of knowledge. Almost all students commented that this effort should be supported, should be integrated into the curriculum, and expanded into other specialties. They would like a wide database of cases to be created in which each student will be able to choose a suitable virtual patient case because semantic markup will be available for easy retrieval and reuse of virtual patients.

## Discussion

### Context

Virtual patients cannot be considered a direct substitute for interpersonal experiential forms of learning, such as real clinical experience. However, their capacity for immediacy and safety has established them as standard learning tools in medical education [46]. There is ample literature available to cover details such as connecting specific clinical guidelines in the design of virtual patients [47] and providing quality control metrics for assessing them [48]. In fact, with the development of a formal MedBiquitous Virtual Patient international standard in 2010 [41,49], virtual patients are a widely available tool in the medical curriculum (lectures, exams, project PBL, synchronous or asynchronous e-learning sessions) [7]. This advent of virtual patients has triggered research for highly specialized, context-specific virtual patient design models for catering to specific medical specialties [50]. In this environment, the capacity of transferring and reusing virtual patient resources into different contexts, for different educational goals, across media and platforms becomes an attainable research goal.

The overall repurposing initiative of educational content has come of age for some time now [51]. With data standard infrastructure established as early as 2002 through the LOM standard [52] followed by the Sharable Content Object Reference Model (SCORM) [53] and the Healthcare LOM schema [54], an effort was initiated in the form of the mEducator project. Both Web 2.0 mash-up technologies and federated, semantic, Web service-based learning content management systems were explored as possible avenues of standardizing the repurposing of medical education content [55]. The overarching purpose was to make educational content discoverable and context-independent to facilitate its reusability and repurposing for different educational goals and across different educational environments [51].

### Contribution of This Work

This work, initially named LinkedLabyrinth during the mEducator project [56], is an extension of the OpenLabyrinth Virtual Patient creation and deployment platform. This extension added semantic annotations and repurposing capacities to the OpenLabyrinth platform [22]. Post-mEducator project activities were focused on aligning efforts with the MedBiquitous Consortium (an organization producing standards for digital health education) [41]. MedBiquitous has adopted the previously described mEducator proposal for the semantic extension of its current standards as evidenced by ongoing technical discussion taking place in its Technical Committees and Working Groups [57]. These developments together with the extension for OpenLabyrinth makes mEducator 3.0 accessible to a completely new user base, that of the OpenLabyrinth platform, and overcomes the lack of standardized sharing mechanisms.

The results presented in this work from the multifaceted assessment of the OLabX provide some insights into the system’s characteristics. First among the system’s strengths is the core repurposing capacity. By using semantic annotation of virtual patient content through the mEducator 3.0 metadata schema in combination with the specifications of the MedBiquitous Virtual Patient standard, OLabX provides a systematic and organized capacity for content repurposing across several axes (context, educational objectives, and platforms).

This assessment also demonstrated this prototype’s weaknesses. At present, the project has built a semantic infrastructure for enabling mEducator 3.0 capabilities. The lack of uptake of this semantic infrastructure is recognized, as demonstrated by weaknesses referred in the results of the experts’ evaluation. The concerns regarding usability are expected because this is a rather unpolished prototype product. Users of the platform would make better use of semantic annotation when more clearly informed of the underlying semantic annotation process and functionality. This interesting anecdotal result touches on the expectations of users that are starting to become semantically aware in the Web 3.0 environment. On the main exploratory track, however, this lack of uptake for the provided services is not unexpected because of the prototype nature of the development of the OpenLabyrinth extensions. These are important follow-up steps to the core effort, which is the implementation of the mEducator semantic infrastructure within the OpenLabyrinth platform.

Furthermore, the evaluation consisted of a series of evaluation episodes performed during various workshops that were held as part of the mEducator project and was not fully designed from the beginning of the assessment procedure. That could be mentioned as a limitation of our study protocol, although it ended up providing us with valuable feedback for the OLabX performance in virtual patient retrieval, discovery, and repurposing. Regarding usability, the 2 trial and evaluation episodes, although they were not preplanned, provided similar SUS scores with only a 10% difference (30% and 40%, see Figures 4 and 5). Additionally, qualitative results appeared to converge across evaluation episodes. Both expert reviewers and evaluating students provided positive feedback for the capacity of OLabX to facilitate meaningful exploration and filtering of virtual patient resources through semantic annotation. They both noted the intuitiveness of the search process and the contribution to it of the extensive metadata scheme that was incorporated in this extension of the OpenLabyrinth platform. The results obtained match those that have arisen from previously published studies as far as fostering of clinical reasoning and students’ virtual patient adoption into the curriculum are concerned [26-28].

The input from the evaluation has already led to a more powerful annotation subsystem for the next version of OpenLabyrinth v3.3 [58]. This new extension is not monolithic. Rather, it is composed of many smaller and flexible submodules, allowing system administrators to enable functionality depending on their institution’s needs. The new functionality includes out-of-the-box custom semantic metadata fields, support for any classification represented in the RDF format, and visual reports with iterative development pending the results of follow-up assessment [59,60].

### Future Directions

Semantic Web technology provides a powerful opportunity to structure and annotate information about virtual patients to allow for easy retrieval, reuse, and exchange of virtual patients’ content between different systems. The current work directly addresses the needs for sharing, exchanging, searching, and cataloging these resources through the mEducator approach [51]. Beyond the resource-level annotation, Semantic Web technology can also be facilitated in the included content element level, indexing the resources in a finer manner. A more sophisticated approach could include metadata about the real-time interaction of students with the content.

Extending this view, there are initiatives that are using this kind of annotation to facilitate the creation of massive open online courses and small OERs [61]. In medical education, the virtual patient space has become quite prolific [46]; therefore, semantic annotation can be an asset in repurposing content and also context or platform [41]. Research in open education has identified that resource and system interoperability are significant aspects for successful endeavors in open education initiatives [62]. The repurposing of virtual patients becomes a significant contributor to open access medical education. On enhancing the efficacy of the latter, the use of virtual patients and PBL in teaching clinical decision making is currently considered a substantially effective instructional approach [46]. The availability of systems such as the one presented in this paper is important especially when one considers curricula adaptations/transformations such as those under development in 6 institutions of the ePBLnet consortium, a European Commission-funded program [63].

Game-based medical education content that allows for more exploratory freedom to the learner provides a different field for case-based content repurposing. Such efforts include the dynamic patient simulator [64], a more open-ended virtual case software; the virtual standardized patient, a more psychologically realistic approach to doctor-patient encounters [65]; or multi-user virtual environment-deployed virtual patients [41,66] for providing a more graphically rich experience for learners. These efforts point to a clear direction of emergent, experiential, dynamically created content in which repurposing capacities enabled by semantic annotations such as those offered through the OLabX platform can provide a method for rapid and even automated content creation and customization.

There are already documented efforts for providing educational resources to medical students through nonconventional hardware (eg, personal data assistants, mobile phones) with encouraging results from the assessment [67]. A repurposing platform that could aggregate and push frequently accessed content from virtual patient usage, along with “tweetations” [68] that reference or push useful resources to mobile devices could allow for a migration of “educemiology” (an educational content proliferation patterns study) [69,70] to the virtual patient space to assess and promote educational content relevant to the specific needs of a learner or group. This emergent semantification of resources through social tagging is not new. The term FolksOntology was coined as early as 2007 [71] to describe the emergence of informal ontologies through social tagging (folksonomies). Efforts for promotion of trending educational resources that are rapidly repurposed for context by the learners themselves would enable this next synergy of Web 2.0′s folksonomies [72] and Web 3.0′s semantics [73] and this could evolve into a custom, localized, and emergent educational content commons. In that context, virtual patients that facilitate experiential anchoring of previously acquired formal knowledge through game-based scenarios could find uses through push notifications. These trending tweetations could be accessed from self-directed learning endeavors instead of authoritative suggested cases. For that purpose, the capacity to rapidly develop virtual patient content by repurposing existing cases, even by allowing learners to customize context according to their special needs instead of just expecting authors to create new cases, could contribute far greater value than that of an ease-of-life tool for virtual patient authors. It would provide an infrastructure for enabling learners and educators to create and consume educational content on a “just-in-time” basis [74], enabling rapid and ubiquitous dissemination of medical knowledge.

## Abbreviations

AUTH: Aristotle University of Thessaloniki
BPN: Best Practice Network
ICT: information and communication technology
IPR: Intellectual Property Rights
LRM: Learning Resource Metadata
MeSH: Medical Subject Headings
NCBO: National Center for Biomedical Ontology
OER: open educational resource
RDF: Resource Description Framework
SUS: System Usability Scale
